# Genetic Parameters of the Growth Rate, Survival Rate and Feed Efficiency Ratio of Turbot (*Scophthalmus maximus*) at an Early Growth Stage

**DOI:** 10.3390/ani15162424

**Published:** 2025-08-19

**Authors:** Donghui Gou, Yilin Wang, Xinan Wang, Zhibin Sun, Aijun Ma

**Affiliations:** 1 State Key Laboratory of Mariculture Biobreeding and Sustainable Goods, Yellow Sea Fisheries Research Institute, Chinese Academy of Fishery Sciences, Qingdao 266071, China; 15713356921@163.com (D.G.); 18254378596@163.com (Y.W.); wangxa@ysfri.ac.cn (X.W.); sunzb@ysfri.ac.cn (Z.S.); 2 China-ASEAN Belt and Road Joint Laboratory on Mariculture Technology (Qingdao), Qingdao 266071, China; 3 Laboratory for Marine Biology and Biotechnology, Qingdao National Laboratory for Marine Science and Technology, Qingdao 266071, China

**Keywords:** turbot, growth rate, survival rate, feed efficiency ratio, heritability, genetic correlation

## Abstract

Genetic improvement, including improving the growth rate, survival rate and feed efficiency ratio (FER), can maximize the production efficiency of turbot. Genetic evaluations of the growth rate, survival rate and FER are required for determining the practicability of including these three traits in a breeding programme. In this study, 20 full-sib families were produced. A method involving a small sample was used to calculate the growth rate, survival rate and FER to represent the corresponding data of an individual. Then, the genetic parameters of the three economic traits were evaluated. The heritabilities for the survival rate, growth rate and FER in turbot were 0.109001, 0.232335 and 0.101866, respectively. The heritabilities of the survival rate and FER were low, while that of the growth rate was moderate. The phenotypic correlations between the survival rate and growth rate, the survival rate and FER, and the survival rate and FER were 0.0919, 0.4609 and 0.2472, respectively. The genetic correlations between the growth rate and survival rate, the survival rate and FER, and the growth rate and FER were 0.0984, 0.3732 and 0.5654, respectively. Apart from a low phenotypic and genetic correlation between the survival rate and growth rate, the correlations among the other traits were moderate.

## Simple Summary

Establishing the genetic variation in breeding target traits is a prerequisite for a successful breeding programme. Thus, estimating the genetic parameters for breeding traits is an essential element of fish breeding programmes. To genetically improve the growth rate, survival rate and feed efficiency ratio of turbot, a genetic evaluation of these three traits is required. Genetic evaluations of the growth rate, survival rate and feed efficiency ratio in turbot showed that the heritabilities of the feed efficiency ratio and survival rate were low, while that of the growth rate was moderate. Apart from a low phenotypic and genetic correlation between the survival rate and survival rate, the correlations among the other traits were moderate. The findings of this study provide background information for determining the optimal strategy for selecting for genetic improvement, including the growth rate, survival rate and feed efficiency ratio, in turbot.

## 1. Introduction

The ultimate goal of selective breeding is obtaining greater economic benefits [1]. Often, within an aquaculture breeding programme, survival and growth rate, which determine the total harvest yield, are extremely desirable economic traits [2,3]. In addition, feed efficiency (FE) plays an important role in delivering economic benefits. The published literature shows that the cost of feed accounts for 30–70% of the total production costs in aquaculture [4]. Therefore, improving FE is key for lowering production costs and achieving sustainability for the aquaculture industry. At present, a considerable amount of research has been carried out on nutritional and husbandry methods for improving FE but little work has been carried out on the use of genetics [4]. In addition, producing fish has an impact on the environment, which creates the need for breeding programmes that decrease these impacts [5,6]. A few studies have already examined the environmental impacts of breeding traits in fish production [7,8]. Studies have shown that improving the feed efficiency ratio (FER; the ratio of body weight (BW) gain over feed intake) can improve profit and decrease environmental impact simultaneously. Therefore, the FER should be included in fish breeding programmes [6].

However, unlike terrestrial livestock, the FER is usually not evaluated in fish breeding programmes because fish are typically raised in water and in large groups, and individual feed intake cannot be exactly measured in group-reared fish [4,6,9]. Nevertheless, considering that production efficiency can be maximized by developing fish with a high survival rate, growth rate and FER [10], genetic improvement research including these three important economic traits still attracts attention. Establishing the genetic variation in the selected traits is a prerequisite for a practical genetic improvement programme [1,11,12,13]. Thus, estimates of genetic parameters for the breeding traits are fundamental in the process of fish breeding [1,14,15]. Therefore, it is important to calculate the FER and then genetically evaluate the three important economic traits. Because the FER is the ratio of BW gain to feed intake, the crucial issue when measuring the FER is to measure both growth and feed intake. Although growth is easily measurable, measuring the feed intake of individual fish is much more challenging [4].

One method for measuring feed intake of fish is to raise the fish in a group and calculate the FE of the group [4,16,17,18,19,20]. Using separately reared full-sib families, it is possible to estimate the genetic variability in each trait [4,10,16,21]. The main advantage of this method is that it enables an estimation of the genetic variation between families [21]. However, this method does not explore the intra-familial variations, resulting in overestimated heritabilities [22,23]. Another method for calculating an individual’s FER is through raising fish separately in culture ponds. This method requires a considerable number of culture ponds. More importantly, this may have a significant impact on feed intake because social interactions are not taken into account [4]. Studies have shown that the feed intake of some fish measured in isolation will differ from measurements taken in groups [24,25,26]. It would be more accurate if feed intake could be measured for individuals kept in groups [4]. Obviously, the two methods described above have some disadvantages. A more accurate way to measure individual feed intake is still needed. In this study, we used small-sample-size data instead of individuals to solve this problem. That is, each family was randomly divided into small samples, and the growth rate, survival rate and FER of each small sample represented the corresponding data of an individual.

In statistics, a sample size of less than 30 is usually considered small, whereas a sample size greater than 30 is large. The larger the sample size, the more accurate the statistical estimation of the population. In this study, to express the FERs of “individuals”, we do not need a large sample representing the population but small samples that can represent an “individual”. In this study, we first divided the family into small samples for feeding and then calculated the survival rate, growth rate and FER of the small sample to represent the corresponding data of an individual. Then, the genetic parameters of the three economic traits were evaluated. The objective of this study was to provide insights into selection using the genetic parameters of the survival rate, growth rate and FER.

## 2. Materials and Methods

### 2.1. Broodstock and Experimental Family

The turbot broodstocks in this study were sourced from the Tianyuan Aquatic Limited Corporation, Yantai, Shandong, China. In January 2020, a 3-year-old turbot was selected as the parent fish for family construction according to certain criteria. The standards were as follows: complete body, normal colour, healthy and uninjured, active action, strong clustering and active feeding. The selected parental fish were cultivated with artificial compound feed, soft pellet feed and forage fish. The reproduction of the parent fish was regulated by setting light and temperature control conditions. The lamp was installed 1–1.2 m above the fishpond from the water surface. The illumination time increased from 8 h to 16 h, and the water temperature increased from 8 to 14 °C. A nested mating design was employed to produce families: each male was paired with two females, creating paternal half-sib groups and full-sib groups. The standard protocol involved the following steps: First, eggs were collected separately from each female. Next, milt was obtained from the male and split into two aliquots. Finally, each aliquot was used to fertilize the eggs of a single female [26]. A total of 20 full-sib families (10 half-sib families) were produced via artificial insemination.

### 2.2. Larval and Juvenile Rearing

During the period from the hatching of fertilized eggs to the end of the experiment (6 months of age), each family needed to be reared separately, unavoidably leading to environmental differences among families. To ensure the same raising conditions during the larval and juvenile culture periods, various effective measures were taken to standardize both the quantity of fish and the environmental conditions. At the 15th, 30th, 45th and 60th days after hatching, the quantity of fish per family was normalized by randomly sampling 10,000, 5000, 2000 and 1000 individuals, respectively. At 3 months of age, each family was divided into 30 glass tanks (40 cm × 40 cm × 40 cm), and 15 fish were reared in each glass tank until they reached 6 months of age. The environmental conditions were standardized during the larval and juvenile culture periods as follows: a water temperature of 18 °C, salinity of 30–40, an illumination intensity of 500–2000 lx, a pH of 7.8–8.2 and dissolved oxygen >6 mg/L. During the period between the third and sixth months, the above five indices were 15–18 °C, 25–30, 500–1500 lx, pH 7.6–8.2 and >6 mg/L, respectively [27]. During the larval and juvenile periods, the feed series for the artificial rearing of turbot was as follows: rotifer—artemia nauplii—microparticle compound feed. During the period from the third to sixth months, the fish were fed artificial pellet feed. The artificial pellet feed used in this experiment was the “Shengsuo brand” compound pellet feed produced by Shengsuo Fishery Feed Research Centre of Shandong Province, Yantai, China. The raw materials are fish meal, shrimp meal, soybean meal, wheat meal, vitamins (A, D3, E, K3, B12, C, etc.), calcium dihydrogen phosphate, copper sulphate, zinc sulphate, cobalt chloride, ferrous sulphate and manganese sulphate, among others. The constituents of the feed ingredients are crude ash (17%), total phosphorus (1.2%), crude protein (48%), crude fibre (6%), calcium (5%), crude fat (8%), water (12%) and lysine (2.0%).

### 2.3. Data Collection

At the beginning of the experiment, the mean BW of fish in each glass tank was weighed using a digital balance. After each feeding, the residual diets in each glass tank were collected and dried. At the end of the experiment, the number of surviving fish in each glass tank was counted, and the average BW was weighed. Additionally, the amount of feed and the dry weight of the residual diets of each glass tank in the whole experimental period were determined, and the dry weight of the residual diets was converted into the weight of the soft pellet feed. Then, the weight gain and feed intake of each glass tank of fish in the whole experimental period were calculated. Based on the results of these procedures, the growth rate, survival rate and FER were calculated using the following formulas: survival rate (%) = 100 × final survival number/initial survival number; growth rate (%) = 100 × (final BW − initial BW)/(final time − initial time) (time unit: day); FER = weight gain (g)/feed intake (g).

### 2.4. Statistical and Genetic Analysis

Each small sample of 15 fish represented an “individual”. The survival rate, growth rate and FER of the small sample represented the corresponding data of an “individual”. The genetic parameters for the survival rate, growth rate and FER were estimated using the following animal model:

*y_i__j_* = *μ* + *a_i_* + *f_j_* + *e_ij_*, (1)

where *μ* = the mean value of the population; *y_ij_* = the observations of each trait (growth rate, survival rate and FER); *a_i_* = the random additive effect of fish; *f_j_* = the random effect of full-sib family; and *e_ij_* = the residual effect.

Narrow-sense heritability is the proportion of the total phenotypic variance explained by additive genetic effects. The formula for calculation is as follows:

(2) $$h^{2}\;=\;\sigma_{a}^{2}/(\sigma_{a}^{2}+\;\sigma_{f}^{2}+\;\sigma_{e}^{2})$$

where $\sigma_{a}^{2}$, $\sigma_{f}^{2}$ and $\sigma_{e}^{2}$ are additive variance components, the full-sib family variance component and the residual variance component, respectively.

The phenotypic and genetic correlation between the three economic traits were analyzed based on the phenotype value and breeding value.

Phenotypic correlation (*r_p_*) can be written as

(3) $$r_{p}\;=\;\mathrm{cov}\;(p_{x},p_{y})/\sqrt{\sigma_{p_{x}}^{2}{.\sigma }_{p_{y}}^{2}}$$

Genetic correlation (*r_g_*) can be written as

(4) $$r_{g}\;=\;\mathrm{cov}\;(a_{x},\;a_{y})/\sqrt{\sigma_{a_{x}}^{2}{.\sigma }_{a_{y}}^{2}}$$

where *p_x_* and *p_y_* are measurements of x- and y-economic traits (the growth rate, survival rate and FER) and cov (*p_x_*, *p_y_*) is the covariance of *p_x_* and *p_y_*, while *a_x_* and *a_y_* are the additive genetic effects of x- and y-economic traits and cov (*a_x_*, *a_y_*) is the covariance of *a_x_* and *a_y_*.

The heritability, phenotypic correlation and genetic correlation were estimated using derivative-free restricted maximum likelihood estimation (MTDFREML) [28]. Prior to statistical analysis, outliers were identified through box plots visualization, and the data normality was evaluated using the Shapiro–Wilk test.

## 3. Results

### 3.1. Descriptive Summary of the Growth Rate, Survival Rate and FER

**Figure 1:**
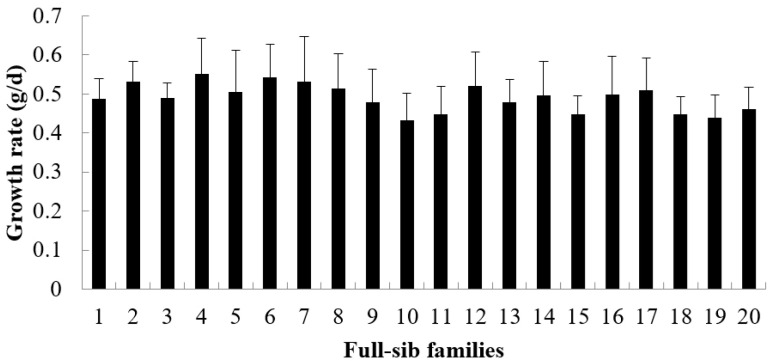
The growth rate of each family from 3 to 6 months (mean value of 30 glass tanks).

**Figure 2:**
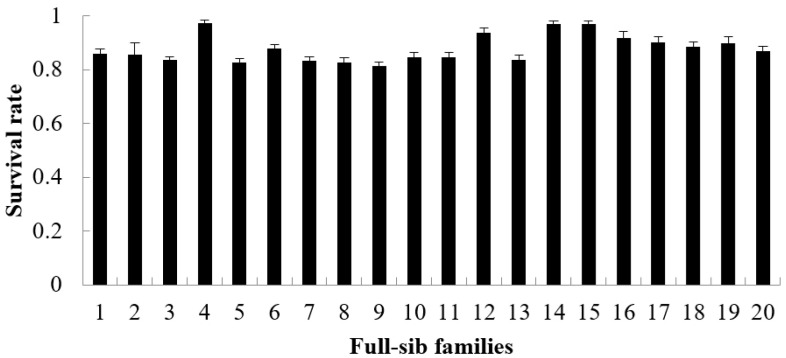
The survival rate of each family from 3 to 6 months (mean value of 30 glass tanks).

**Figure 3:**
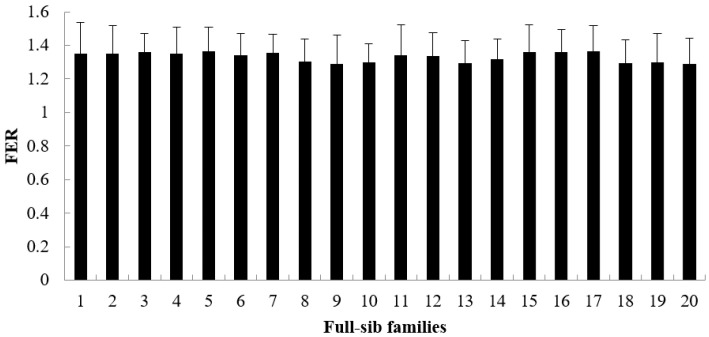
The FER of each family from 3 to 6 months (mean value of 30 glass tanks).

The growth rate, survival rate and FER are showed in Figure 1, Figure 2 and Figure 3. During the period from 3 to 6 months of age, the growth rate values of 20 families ranged from 0.4327 to 0.5511 (Figure 1), the survival rate values ranged from 0.8135 to 0.9727 (Figure 2) and the FER values ranged from 1.2886 to 1.3635 (Figure 3). The Shapiro–Wilk normality test verified that all datasets followed a normal distribution.

### 3.2. Genetic Evaluation for the Growth Rate, Survival Rate and FER

The variance component and the heritabilities for the growth rate, survival rate and FER are presented in Table 1. We found that the heritability was 0.232335 for the growth rate, 0.109001 for the survival rate and 0.101866 for the FER. Therefore, the heritabilities of the survival rate and FER were low, while that of the growth rate was moderate.

Phenotypic and genetic correlations for the survival rate, growth rate and FER are shown in Table 2. The phenotypic correlations between the survival rate and growth rate, growth rate and FER and survival rate and FER were 0.0919, 0.4609 and 0.2472, respectively. The genetic correlations between the survival rate and growth rate, the growth rate and FER and the survival rate and FER were 0.0984, 0.5654 and 0.3732, respectively. Apart from the low phenotypic and genetic correlations between the growth rate and survival rate, the other correlations were moderate.

## 4. Discussion

Direct selection for FE in fish is difficult because it is not easy to measure it directly [4,9]. A better method is to determine traits highly correlated with FE and to improve the FE of fish via indirect selection [4,29]. Therefore, it is greatly significant to formulate a breeding plan for the FE by genetically evaluating the FE and main economic traits of fish. The selection potential of feed intake in rainbow trout (*Oncorhynchus mykiss*) was estimated by measuring individual daily feed intake (DFI). Heritability for the average DFI was low (0.10). Phenotypic and genetic correlations between DFI and BW ranged from 0.48 to 0.54 and 0.72 to 0.90, respectively. Phenotypic and genetic correlations between DFI and daily weight gain (DG) ranged from 0.51 to 0.74 and 0.86 to 0.96, respectively [23]. The heritability and genetic correlation between the survival rate, growth rate and feed conversion efficiency in rainbow trout were estimated. Heritability was very low for the survival rate, BW and feed conversion efficiency. The genetic correlations between the FER and BW ranged from 0.63 to 0.99 [10]. To assess the genetic potential for selecting increased FE in rainbow trout, the heritabilities and correlations for BW, DG and DFI were estimated. The average heritabilities for BW and DG relative to diets at different fish ages were 0.28 and 0.33, respectively. Average heritability for DFI relative to diets at different fish ages was low (0.10). On a diet with normal protein levels, the genetic correlations between DG, BW and DFI were very strong. On a high-protein diet, the corresponding genetic correlations were low to moderate [30]. Genetic parameters for food conversion efficiency, growth and food consumption in young rainbow trout were estimated using 231 full-sib family groups. The mean heritabilities estimates were 0.03 for food conversion efficiency, 0.26 for growth, and 0.41 for food consumption. Phenotypic and genetic correlations were 0.96 and 0.99 between growth and food consumption, 0.72 and 0.80 between food conversion efficiency and growth, and 0.53 and 0.25 between food conversion efficiency and food consumption [31]. Genetic parameters for feed intake and BW in European whitefish (*Coregonus lavaretus* L.) were estimated. Heritabilities for feed intake and BW ranged from 0.05 to 0.16 and 0.06 to 0.22, respectively. Phenotypic and genetic correlations between feed intake and BW ranged from 0.86 to 0.88 and 0.93 to 0.97, respectively [32]. Genetic parameters for feed intake, growth and FE in European whitefish (*Coregonus lavaretus* L.) reared with soybean meal and fishmeal diets were estimated. In both diets, DG and DFI showed moderate heritability (heritabilities for DG were 0.20 and 0.26, respectively, and heritabilities for DFI were 0.17 and 0.23, respectively), whereas FE exhibited highly low heritability, with estimates not significantly different from zero (heritabilities of FE, 0.07 and 0.06, respectively). Genetic and phenotypic correlations between DG and DFI were highly significantly positive in both fishmeal (R_g_ = 0.97 and R_p_ = 0.88) and soybean meal diets (R_g_ = 0.93 and R_p_ = 0.86) [33].

An important issue regarding selective breeding for FE is the choice of FE index [4,34]. In this study, the FER was used as the evaluation index of the FE, and genetic evaluations of the FER, survival rate and growth rate were carried out. The heritabilities for the FER, survival rate and growth rate in turbot were 0.101866, 0.109001 and 0.232335, respectively. Therefore, the heritabilities of the survival rate and FER were low and the heritabilities of the growth rate were moderate. The phenotypic correlations between the survival rate and growth rate, the growth rate and FER and the survival rate and FER were 0.0919, 0.4609 and 0.2472, respectively. The genetic correlations between the growth rate and survival rate, the growth rate and FER, and the survival rate and FER were 0.0984, 0.5654 and 0.3732, respectively. Apart from the low phenotypic and genetic correlations between the growth rate and survival rate, the other correlations were moderate. Based on these results and the research on rainbow trout and European whitefish mentioned above, we conclude that FE heritability estimates are invariably low, regardless of species or experimental method [4,30]. The heritability of the FER was similar. The results on genetic and phenotypic correlations between the FER and economic traits obtained from this study were lower than those in rainbow trout [7,23,31] and European whitefish [32,33]. It is speculated that these differences were mainly attributed to the fish species selected, the different developmental periods of samples, the different diets, the different evaluation indexes for FE, the different sample sizes, and the different statistical models used.

For selective breeding in aquaculture, breeding new varieties with multiple good target traits is the main objective of genetic improvement [7,35,36,37,38]. At present, two methods are used to achieve this breeding goal. One is traditional cross-breeding, while the other is a multi-trait integrated breeding programme. For multi-trait integrated breeding, the genetic correlation among selected traits should be considered first. Genetic improvement can be carried out using multi-trait integrated breeding technology when there is a positive genetic correlation between the improved traits. The higher the positive genetic correlation among traits, the more significant the breeding effect. When there is a negative genetic correlation between breeding traits, because of the antagonistic effect among traits, a cross-breeding method should be used for genetic improvement [39]. Our research showed that a positive genetic correlation existed among the survival rate, growth rate and FER. Therefore, multi-trait integrated selection should be adopted to simultaneously improve the survival rate, growth rate and FER of turbot. Additionally, genomic tool development for FE could be examined in turbot. Another useful approach involves identifying molecular markers associated with QTL (quantitative trait loci) or metabolic pathways that influence genes regulating feed intake or FE [4,40,41].

## 5. Conclusions

The heritabilities of the survival rate and FER were low, while that of the growth rate was moderate. Apart from a positive and low phenotypic and genetic correlation between the survival rate and growth rate, the correlations among other traits were moderate. Given the positive genetic correlations among the three traits, a breeding method employing multi-trait integrated selection should be adopted to simultaneously improve the survival rate, growth rate and FER of turbot. In addition, the development of genomic tools for the FE could be studied in turbot. Another valuable strategy could be identifying molecular markers associated with quantitative trait loci or metabolic pathways that influence genes controlling feed intake or FE.

## Figures and Tables

**Table 1:** The variance components and heritability (*h*^2^) of three economic traits in turbot.

Economic Traits	Variance Components			
	$\sigma_{a}^{2}$	$\sigma_{f}^{2}$	$\sigma_{e}^{2}$	*h* ^2^
Growth rate	0.000411	0.000016	0.001342	0.232335
Survival rate	0.000310	0.000047	0.002487	0.109001
FER	0.000131	0.000018	0.001137	0.101866

**Table 2:** The phenotypic (*r_p_*, above the diagonal) and genetic (*r_g_*, under the diagonal) correlations of three economic traits in turbot.

Economic Traits	Growth Rate	Survival Rate	FER
Growth rate		0.0919 ± 0.02344	0.4609 ± 0.0470 **
Survival rate	0.0984 ± 0.0133		0.2472 ± 0.0133 **
FER	0.5654 ± 0.0896 **	0.3732 ± 0.0755 **	

** The correlation is significant at the 0.01 level (two-tailed).

## Data Availability

The datasets analyzed are available from the corresponding author on reasonable request.
